# Explanation of chronic stressors in older women with uterine fibroids—a qualitative study

**DOI:** 10.3389/fpubh.2025.1652346

**Published:** 2025-10-02

**Authors:** Yanzi Guo, Xinyue Xie, Lishi Huang, Kechen Fan, Congcong Yu, Dongjian Yang, Nan Tuo

**Affiliations:** ^1^The International Peace Maternity and Child Health Hospital, School of Medicine, Shanghai Jiao Tong University, Shanghai, China; ^2^Shanghai Key Laboratory of Embryo Original Diseases, Shanghai, China

**Keywords:** qualitative study, content analysis, older women, uterine fibroids, chronic stressors

## Abstract

**Introduction:**

Older women with uterine fibroids experience complex challenges that extend beyond physical symptoms to include emotional stressors and other stressors. This study aimed to explain the chronic stressors faced by older women with uterine fibroids, with a focus on their effects on physical health, emotional well-being and daily functioning.

**Methods:**

This qualitative study employed semi-structured, in-depth interviews with 28 women aged 60 years or older diagnosed with uterine fibroids, conducted between 2023 and 2024 in China. Participants were purposively sampled to ensure diversity in education, marital status, and economic background. Each interview lasted approximately 30–40 min, was audio recorded with consent, and transcribed verbatim. Data were analyzed using conventional content analysis with NVivo 14 used for data management. To ensure rigor, Four-Dimensions Criteria (credibility, dependability, confirmability, and transferability) were applied.

**Results:**

The study sample consisted of 28 older women with uterine fibroids, with a mean age of 66.18 ± 4.997 years. The data were categorized into four main stressor categories: “health stressor” (with three subcategories), “family stressor” (with three subcategories), “financial stressor” (with two subcategories), and “social stressor” (with four subcategories). Continued uncertainty about symptom progression exacerbated anxiety, while cultural expectations around family roles deepened feelings of guilt and isolation. Financial limitations further restricted access to care, heightening both physical and emotional distress. The lack of social support exacerbates patients’ sense of insecurity. Moreover, the four categories of stressors were interrelated, with challenges in one domain often compounding or intensifying stress in the others.

**Conclusion:**

This study revealed the multidimensional chronic stressors experienced by older women with uterine fibroids, including challenges in health management, family support, financial stability, and social inclusion. These domains interacted to form a reinforcing network that intensified both physical and psychological burdens. Addressing these interconnected stressors through integrated medical, psychosocial, and policy strategies is essential to improving the well-being of this population.

## 1 Introduction

As the global population ages, the health issues of older women have increasingly attracted social attention. These challenges encompass not only common chronic conditions such as hypertension, diabetes, osteoporosis, and cardiovascular diseases but also those arising from physiological decline and hormonal changes (1–4). For instance, many older women continue to experience persistent pelvic pain, frequent urination, constipation, and other symptoms post-menopause, which are often linked to gynecological conditions such as uterine fibroids (5). Moreover, these health concerns are frequently accompanied by psychological and social pressures, particularly loneliness, depression, and caregiving burdens. Together, these factors severely impact the quality of life for older women and place significant strain on global healthcare systems.

Uterine fibroids, also known as uterine leiomyomas, the most prevalent benign tumors of the female reproductive system, are associated with hormonal growth and development. The incidence of pathologically diagnosed fibroids rises with age, peaking at 50 years (6). Uterine fibroids can manifest in various physical symptoms, including pelvic pain, heavy menstrual bleeding, frequent urination, and constipation (7). Age is a key risk factor for the development of fibroids due to their hormone-dependent nature. Fibroids typically develop after menarche and continue to progress during the reproductive years (8). In premenopausal women, the median growth rate of fibroids is 9% every 6 months (9). While many fibroids may regress post-menopause, subset of women do not experience fibroid regression after menopause, some lesions persist or even grow (10, 11), sustaining mass-effect symptoms into later life and thereby sustaining anxiety and health-care use. In older women symptomatic uterine fibroids are predominantly associated with bulk-related discomforts rather than heavy cyclic bleeding. Typical bothersome manifestations include pelvic pressure or abdominal fullness, low back pain, and bowel and bladder dysfunction such as urinary frequency/urgency and constipation due to compression of adjacent organs. These symptom clusters are well documented in reviews and clinical summaries of uterine leiomyomata (12, 13). In rare cases, postmenopausal spotting or bleeding that may raise concern for malignancy (14).

Epidemiologically, uterine fibroids remain both common and clinically relevant in later life. Global syntheses indicate that by about age 50, up to 70–80% of women have fibroids detectable by imaging or histology (15), although only 20–50% become clinically symptomatic or require treatment (16). In China, burden analyses drawing on the Global Burden of Disease framework show rising incidence, prevalence, and disability (DALYs) of uterine fibroids from 1990 to 2019, with risk increasing with age; while overall prevalence typically peaks before menopause, a non-trivial residual burden persists beyond menopause (17). With longer life expectancies and advances in diagnostic imaging (18), an increasing number of postmenopausal women are being diagnosed with uterine fibroids. Across studies of uterine fibroid disease in older women, these symptom burdens are linked to decrements in health-related quality of life, including pain, limitations in daily activities and sleep, and psychological distress. Older women with fibroids often face a complex intersection of medical, psychological, and social stressors that complicate the management of their health, which underscores the need to understand and address the lived impact of fibroids in older women.

Chronic stress refers to the physiological or psychological response triggered by prolonged internal or external stressors (19). Whether the stressor is physically present or recalled, it elicits the same response, activating the body’s chronic stress mechanisms (19). Chronic stressors are typically linked to long-term problems, conflicts, and threats encountered in daily life (20). These stressors, including neighborhood environment, financial strain, interpersonal conflict, work-related pressure, and caregiving, have been identified as significant contributors to disease and mortality (21). The body’s response to chronic stress involves remaining in a heightened state of alertness, even when no immediate danger is present. Individuals may experience symptoms such as anxiety, depression, sadness, anger, irritability, social isolation, headaches, menstrual issues, abdominal pain, back pain, muscle discomfort, and difficulty concentrating (22). For older patients with uterine fibroids, chronic stress may stem from a combination of physical discomfort, limited treatment options, and the broader effects of aging on health and daily functioning. In the context of chronic illness, stress is further intensified by the enduring nature of the condition, feelings of helplessness, and uncertainty about disease progression.

Lazarus and Folkman’s stress and coping theory (23) suggests that stress arises when individuals perceive that the demands of a situation exceed their coping resources, leading to psychological and physiological strain. Chronic stressors, such as persistent illness, financial strain, or family conflict, continuously disrupt this balance, thereby affecting health and well-being. In addition, the biopsychosocial model (24) highlights that health outcomes are shaped not only by biological symptoms but also by psychological states and social environments, providing a broader perspective for understanding stress in chronic illness. These theoretical perspectives together provide a foundation for examining the chronic stress experienced by older women with uterine fibroids, whose illness often interacts with physical, social, and financial challenges.

While these classic frameworks provide valuable foundations, they remain limited in explaining the full range of stressors encountered by older women with uterine fibroids. Lazarus and Folkman’s stress–coping model highlights the imbalance between perceived demands and coping resources (25), but it largely focuses on individual cognitive appraisal. Similarly, the biopsychosocial model emphasizes multidimensional influences on health (26), yet it does not sufficiently capture culturally embedded stressors, such as intergenerational guilt (27), or institutional factors, including the hospital environment and doctor-patient communication barriers (28). These gaps underscore the necessity of constructing a more detailed theoretical lens. This study seeks to explain the chronic stressors of older women with uterine fibroids by extending classic models to incorporate sociocultural and healthcare system dimensions, thereby generating a more comprehensive understanding and advancing both theory and practice in women’s health research.

Substantial quantitative studies have provided valuable insights into the epidemiology, risk factors, and clinical management of uterine fibroids (15, 29), they offer limited understanding of the subjective experiences of older patients. Such approaches often fail to capture the subjective dimensions of illness, including emotional burdens, social isolation, and family-related stressors (30), which are particularly salient in older women. Qualitative research, by contrast, allows for in-depth exploration of these complex experiences (31), revealing how symptoms intersect with psychological, social, and cultural contexts. However, these dimensions remain underexplored, particularly among older patients. As the global aging population expands, addressing these factors is crucial for enhancing the quality of life for older individuals living with uterine fibroids.

This study aims to explain the chronic stressors experienced by older patients with uterine fibroids through qualitative research. The primary goal is to determine key stressors affecting the health and well-being of these patients, as well as to explore how these stressors influence their daily lives. By gaining a deeper understanding of the reported experiences of older women with uterine fibroids, this research seeks to inform the development of more comprehensive care strategies that address both the physical and psychosocial needs of this vulnerable population.

## 2 Material and methodologies

### 2.1 Aim

This study aimed to explore the chronic stressors experienced by older women with uterine fibroids and their impact on daily life.

### 2.2 Study design

This was a qualitative study based on conventional content analysis raised by Graneheim and Lundman (32), chosen to inductively derive subcategories and categories from participants’ narratives without relying on preconceived frameworks.

### 2.3 Participants

This study explored the experiences of older women with uterine fibroids. The semi-structured interviews targeted Chinese women aged 60 or older, diagnosed with uterine fibroids, with illness durations ranging from 1 month to 20 years. Participants had no history of psychiatric disorders, were lucid, and expressed a willingness to participate in the study and share their stressors. Participants were recruited from a sample of older women attending the International Peace Maternity and Child Health Hospital, School of Medicine, Shanghai Jiao Tong University (IPMCH) between November 2023 and May 2024. We adopted purposive sampling with maximum variation to ensure diversity in illness duration, education level, marital status, and financial status, resulting in a final sample of 28 women.

### 2.4 Data collection procedure

Data for this study were collected through face-to-face semi-structured in-depth interviews conducted in 2024, in a calm and comfortable environment. The interviews commenced with broad questions, such as “What stressors has fibroids caused you and how do you cope with it?” followed by exploratory prompts like “Can you expand on that?,” “Tell me about your feelings; can you provide an example?,” and “How do you cope?.” This approach allowed for the identification of potential areas of concern. Each interview lasted approximately 30–40 min and was recorded using a digital voice recorder. Purposive sampling continued until data saturation was reached—when no new information emerged and the data corroborated previous findings. A total of 31 interviews were conducted with 28 participants, and transcripts were written up after each session.

### 2.5 Data analysis

We applied conventional content analysis following the method of Graneheim and Lundman. First, transcripts were read repeatedly to gain an overall understanding. Meaning units were then identified, condensed, and coded. Codes with similar content were grouped into subcategories, which were further abstracted into categories. Data collection and analysis proceeded concurrently to allow constant comparison. To facilitate data management, NVivo software (version 14) was used.

### 2.6 Rigor

To ensure trustworthiness, we applied the four criteria of credibility, dependability, confirmability, and transferability. Credibility was enhanced through prolonged engagement with participants, member checking, and peer debriefing within the research team. Dependability and confirmability were supported by maintaining an audit trail and having the coding process reviewed by external experts. Transferability was achieved through purposive sampling with maximum variation.

### 2.7 Ethical considerations

Ethical considerations were strictly followed to ensure the protection and respect of participants’ rights and well-being throughout the study. Ethical approval was obtained from the IPMCH Ethical Review Committee (approval number: GKLW-A-2024-011-01) prior to the study’s initiation. All participants were thoroughly informed about the study’s purpose, procedures, potential risks, and their right to withdraw at any time without consequence. Written informed consent, including explicit permission to record audio during the interviews, was obtained from each participant prior to data collection. Confidentiality and anonymity were strictly ensured.

## 3 Results

The study participants comprised 28 older women diagnosed with uterine fibroids, with a mean age of 66.18 ± 4.997 years. Of the participants, 89.29% were married, while 10.71% were divorced or widowed, no participants reported never being married. Regarding education, 57.14% had completed high school or higher, while 42.86% had completed junior high school or less. The duration of uterine fibroid symptoms ranged from 1 month to 20 years. Notably, 46.43% of the participants had diabetes, and 53.57% had hypertension. In terms of body mass index (BMI), 50% of the participants had a normal BMI, 35.71% were classified as overweight, and 14.29% were obese (Table 1).

**Table 1:** Study participant characteristics.

No.	Age	Occupation	Education background	Course of illness
1	60	Employed	Junior high school	5y
2	63	Retired	Undergraduate	10y
3	71	Retired	Primary school and below	3y
4	61	Retired	Junior high school	1y
5	61	Retired	High school	7y
6	65	Retired	Undergraduate	2y
7	64	Retired	High school	2y
8	65	Retired	Undergraduate	1y
9	63	Retired	High school	5y
10	67	Retired	High school	4y
11	62	Retired	Junior high school	7y
12	62	Retired	College	5y
13	69	Retired	Undergraduate	20y
14	63	Retired	High school	10y
15	69	Retired	Junior high school	2 m
16	66	Retired	Junior high school	7 m
17	72	Retired	High school	9 m
18	61	Retired	Junior high school	15y
19	62	Retired	High school	20y
20	62	Retired	Junior high school	10y
21	79	Retired	High school	10y
22	73	Retired	College	10y
23	72	Retired	Junior high school	1 m
24	70	Retired	Junior high school	15y
25	75	Retired	Junior high school	20y
26	62	Employed	Junior high school	6y
27	70	Retired	High school	20y
28	64	Employed	Junior high school	3 m

A total of 31 interviews were conducted with 28 participants. Three participants (P11, P17, and P23) were interviewed twice to clarify and enrich their accounts.

### 3.1 Qualitative analysis of chronic stressors

According to the principles of conventional content analysis, a total of 89 codes were extracted from the detailed narratives of the participants. These codes were repeatedly reviewed, compared, and grouped based on similarities and semantic relevance. Through this process, the data were organized into 4 overarching categories and 12 subcategories, which comprehensively captured the multidimensional stressors experienced by older women with uterine fibroids. The coding process and examples of abstraction from codes to subcategories and categories are illustrated in Table 2.

**Table 2:** Categories, subcategories, and codes discovered in the study.

Category	Subcategory	Representative codes
Health-related stressors	Symptom control and uncertainty	Persistent symptoms despite menopause Uncertainty about how disease will progress Mismatch between treatment efficacy and patient expectations Anxiety about follow-up results Frustration over slow symptom relief Uncertainty about when to seek surgery
	Fear of malignancy	Anxiety about potential malignant transformation Distrust of “benign” diagnosis Overestimation of hidden cancer risk Frequent requests for reassurance
	Mental health status	Ongoing anxiety and restlessness Feelings of helplessness and vulnerability Emotional exhaustion from long-term illness Minimization of illness by social environment
Family stressors	Lack of caregiving support	Family rarely accompany to hospital visits Absence of children’s daily tasks Feeling emotionally neglected by family members Insufficient understanding of illness from relatives Perception of being a burden within the household
	Intergenerational guilt	Regret over inability to help adult children Conflict between personal health needs and family responsibilities Pressure from cultural expectations of filial piety
	Marital strain and sexual dysfunction	Discomfort and pain during intercourse Reduced marital communication about sexual health Feelings of distance or estrangement from spouse Concerns about being less valued as a partner
Financial stressors	Healthcare cost burden	High out-of-pocket medical expenses due to lack of insurance Repeated follow-up visits increase cumulative costs Delaying or skipping care due to financial strain
	Post-retirement income challenges	Limited pension insufficient for medical needs Reducing frequency of check-ups to save money Balancing daily expenses with medical costs
Social stressors	Work-related anxiety	Fear of losing employment due to illness Pressure to keep working despite poor health Dependence on work for financial stability
	Hospital attendance stress	Anxiety triggered by noisy hospital environments Discomfort with long waiting times Stress from impersonal hospital interactions Anticipation of hospital visits as a source of fear
	Doctor–patient information gap	Difficulty understanding medical terminology Consultations perceived as rushed Lack of tailored information about prognosis Feeling dismissed when asking questions Confusion over treatment recommendations
	Reliance on peer support and informal education	Emotional reassurance from peer interactions Reliance on patient-to-patient communication Use of mass media for disease knowledge Seeking information from online resources

### 3.2 Health-related stressors

Health-related stressors among older patients with uterine fibroids arises from persistent physical symptoms, unpredictable disease progression. This cumulative burden leads to both physical discomfort and psychological distress, particularly when symptom trajectories remain unclear or fluctuate over time.

#### 3.2.1 Symptom control and uncertainty

This subcategory reflects participants’ concerns about unresolved symptoms and the lack of clear medical guidance, which left them unsure about disease trajectory and symptom management. Nine participants (32.14%) reported ongoing anxiety over unresolved symptoms and frustration when expected post-menopausal regression did not occur.

*“The doctor said fibroids might shrink after menopause, but mine haven’t. I hope they can get smaller.”* (Participant 5)

“*Maybe I'll end up having my uterus cut out.”* (Participant 19)

*“I have most symptoms of fibroids, but the doctor just said to follow up. It’s hard to feel at ease without treatment.”* (Participant 10)

#### 3.2.2 Fear of malignancy

Despite the benign nature of uterine fibroids, seven participants (25.00%) voiced unwarranted concern about the possibility of cancer, often stemming from a lack of understanding about their condition. The absence of adequate education about the benign nature of fibroids, their growth patterns, and the factors influencing their progression fueled unnecessary anxiety. Fear of malignancy was reported not only by symptomatic patients but also by those without obvious discomfort, who interpreted the absence of symptoms as a potential hidden danger.

*“I don't feel any discomfort from uterine fibroids, which is what worries me the most. I'm afraid it may turn into bad.”* (Participant 4)

*“I’m afraid it may turn into cancer, but I don’t feel anything.”* (Participant 13)

“*The doctor told me it was a benign tumor, but I was still scared. You know, there are so many types of malignant tumors right now.”* (Participant 12)

#### 3.2.3 Mental health status

The psychological impact of living with uterine fibroids extends far beyond physical symptoms, significantly influencing patients’ emotional well-being. Approximately one-third of participants (*n* = 9, 32.14%) participants reported experiencing distress, depression, and emotional exhaustion due to the chronic nature of their condition and the perceived lack of control over their health.

*“Sometimes I just feel so tired—not just physically, but mentally. It’s like this thing is always there, and I can’t escape it.”* (Participant 7)

“*People say, ‘Oh, it’s just fibroids—every woman has them.’ But that doesn’t make the pain any less real.”* (Participant 3)

### 3.3 Family stressors

Family stressors played a significant role in shaping the experiences of older patients, as reported by participants. This stress manifested in three interconnected dimensions: care deficits, intergenerational role conflicts, and marital intimacy tensions. These stresses were further intensified by persistent cultural expectations surrounding family contributions, particularly as older patients’ health declined due to uterine fibroids.

#### 3.3.1 Lack of caregiving support

Fifteen participants (53.57%) highlighted the logistical and emotional gaps in family caregiving. Societal work pressures often limit the ability of family members to act as primary caregivers. While older patients frequently rely on family support, the reality is that caregivers are often stretched thin by work commitments and other responsibilities. This situation results in feelings of isolation and abandonment, as patients may feel that their health concerns are not being prioritized. In many cases, patients must navigate healthcare systems independently, which is both physically and emotionally taxing.

*“My daughter has little annual leave, so she rarely accompanies me.”* (Participant 5)

“*The child said that he was too busy at work and wanted me to seek medical attention alone.”* (Participant 9)

Emotional strain also arises when caregivers fail to empathize with the patient’s struggles or lack a deep understanding of the medical condition. When caregivers express frustration or impatience, it further amplifies the patient’s sense of isolation and helplessness.

*“My children are impatient when discussing treatment options.”* (Participant 7)

“*Maybe I'm overly worried, and my family doesn't feel the need to be so nervous. They couldn't understand my feelings.”* (Participant 24)

“*I don't feel any emotional support from my family.”* (Participant 27)

#### 3.3.2 Intergenerational guilt

Twelve participants (42.86%) reported feeling guilty for not being able to fulfill traditional family roles, such as providing practical assistance. This sense of guilt, where older parents perceive themselves as burdens rather than contributors, can significantly affect their mental and emotional well-being. In many cultures, particularly in China, where family interconnectedness is a core value, older parents are expected to support younger generations. The inability to meet these expectations can lead to feelings of inadequacy and depression.

*“It’s a mum’s responsibility to give her child more support.”* (Participant 20)

*“Due to ill health, I am now unable to care for my grandchildren as I was able to do.”* (Participant 14)

#### 3.3.3 Marital strain and sexual dysfunction

Chronic symptoms related to uterine fibroids, such as pelvic pain and, in some cases, abnormal uterine bleeding or occasional postmenopausal spotting reported by participants, often disrupt intimacy and lead to marital conflict. Sexual dysfunction in chronic illness can create a cycle of relational stressor, where physical limitations exacerbate emotional and interpersonal strain. For many patients, the inability to maintain a normal sexual relationship contributes to feelings of guilt, frustration, and emotional withdrawal, which further strains the marriage. These emotional burdens can spill over into other areas of the relationship, often resulting in arguments or a sense of disconnection.

*“The discomfort in the lower abdomen makes me unable to have sex.”* (Participant 1)

“*Bleeding makes intercourse impossible—I feel like failing as a wife.”* (Participant 18)

“*Sex has become a burden, not a connection.”* (Participant 15)

### 3.4 Financial stressors

Financial instability, primarily driven by healthcare costs and limited post-retirement income, was a significant stressor for older patients. The combination of fixed incomes and rising medical expenses created an overwhelming financial burden, often limiting access to necessary care.

#### 3.4.1 Healthcare cost burden

Participants without insurance faced severe financial stress due to out-of-pocket medical expenses. For older patients, especially those lacking sufficient insurance coverage, the cost of medical treatment can be prohibitive. As healthcare costs continue to rise, many older patients are forced to make difficult choices about their care, often having to prioritize essential treatments over other basic needs, such as food and housing.

*“I pay for treatment myself because I lack social insurance."* (Participant 16)

“*I didn’t have a steady job, and I have less money for health insurance, and now it costs me some money to go to the doctor.”* (Participant 8)

#### 3.4.2 Post-retirement income challenges

Fixed incomes after retirement pose a significant challenge for patients in affording ongoing medical treatment. The shift to a fixed income can create substantial financial strain, especially when unexpected healthcare costs arise. For older patients, the need to ration care due to financial constraints can lead to worsened health outcomes over time. These patients may delay or forgo necessary treatments to avoid financial strain, which can increase the severity of their condition.

*“With lower income after retirement, the financial pressure is overwhelming.”* (Participant 14)

“*Retirement is already a low income, and it costs money to go to the doctor.”* (Participant 11)

“*I am now reducing the frequency of my follow-up appointments to cut down on overheads.”* (Participant 22)

### 3.5 Social stressors

Participants’ accounts determined four primary areas of social stressors for older patients with uterine fibroids: workplace difficulties, pressures in the healthcare environment, barriers to doctor-patient communication, and a lack of social support. These stresses highlight the social system’s neglect of older individuals’ needs, with many patients continuing to work despite deteriorating health to maintain financial stability; the noisy, overwhelming hospital environments exacerbate their anxiety; miscommunication with healthcare providers leaves patients feeling marginalized; and the absence of formal support structures forces patients to rely on informal patient networks. Together, these structural challenges amplify patients’ sense of powerlessness and intensify their social pressures.

#### 3.5.1 Work-related anxiety

Participants who were still employed expressed concerns about the impact of their condition on job performance. For older patients who continue working, there is often anxiety that their health issues will interfere with their ability to complete tasks, potentially leading to job loss. This job insecurity adds another layer of stress, as many older individuals depend on their employment not only for financial stability but also for a sense of purpose.

*“I worry my condition affects performance, because I often feel discomfort in my lower abdomen. I worry that I’ll lose my job.”* (Participant 1)

“*I would like to work for extra income while my body can still hold out.”* (Participant 6)

“*Even though I'm not feeling well, I still don't want to give up my job.”* (Participant 18)

#### 3.5.2 Hospital attendance stress

The clinical environment itself exacerbates stress, with many patients expressing discomfort related to hospital visits. Factors such as long waiting times, noise, and impersonal interactions contribute to an atmosphere of heightened anxiety and stress.

*“The noisy hospital makes me uneasy. Every follow-up requires courage.”* (Participant 17)

“*I have to go to the hospital for a follow-up check after a while, which makes me very anxious. I don’t know when I can finish this check-up.”* (Participant 18)

#### 3.5.3 Doctor-patient information gap

The gap in medical knowledge and communication between healthcare providers and older patients with uterine fibroid emerged as a significant social stressor. Fourteen Participants (50.00%) expressed frustration over insufficient health literacy support, where complex medical terminology was seldom explained in understandable terms, and clinical interactions were often constrained by time, leaving consultations feeling rushed and incomplete. Many also noted a lack of proactive guidance, such as personalized advice on symptom management or long-term prognosis, and a noticeable asymmetry in decision-making, where treatment options were presented without clear explanations of alternatives or associated risks. These communication barriers resulted in inadequate explanations, confusion surrounding care plans, and a pervasive sense of powerlessness in managing their condition.

*“The doctor said ‘asymptomatic fibroids,’ but I did not know what that meant. No one explained whether I should worry or not.”* (Participant 8)

*“Every visit feels like an assembly line. No one has time to listen.”* (Participant 15)

*“I wish doctors would treat me as a partner, not just a case.”* (Participant 21)

*“They told me to ‘wait and watch,’ but why? Does that mean it’s serious or harmless?”* (Participant 3).

#### 3.5.4 Reliance on peer support and informal education

Seventeen participants (60.71%) believed that social support played a pivotal role in coping with the disease. Engaging with others facing similar health challenges provided both emotional support and reassurance. Additionally, participants sought to expand their understanding of the disease by watching relevant TV programs, reading books, browsing trusted websites, and attending community health lectures.

*“Discussing my condition with fellow patients gives me peace of mind.”* (Participant 25)

*“I searched online to learn more about fibroids.”* (Participant 20)

## 4 Discussion

This study aimed to examine the chronic stress experienced by older women with uterine fibroids, utilizing qualitative analysis to uncover the multidimensional stressors impacting their physical, emotional, and social well-being. The results identify several critical stressors, categorized into health-related, family-related, financial, and social stressors. These findings are valuable for healthcare providers and caregivers, offering insights into the specific needs of this population and highlighting areas for targeted support and intervention. The study also emphasizes the need for a holistic approach to care, addressing not only the physical aspects of fibroids but also the emotional and social dimensions that profoundly affect the overall well-being of older women.

### 4.1 Health-related stressors and uncertainty

Health-related stressors were one of the most significant challenges determined by the participants. The uncertainty surrounding disease progression was a major concern. Many women in this study expressed anxiety, often due to a lack of clarity about the potential benefits of treatment and the future trajectory of their condition. In particular, the expectation that fibroids might shrink after menopause did not materialize for many participants, leading to increased frustration and anxiety. This finding aligns with imaging studies showing that some fibroids persist or even grow after menopause, contrary to common assumptions of regression (33).

Additionally, many participants voiced concerns about malignancy despite the benign nature of their condition. This fear is common among chronic illness populations, where patients often struggle to distinguish between benign and malignant conditions (34). Our findings suggest that insufficient understanding of the benign course of fibroids left many participants uncertain about their health, reinforcing fears of malignancy and amplifying anxiety about disease progression.

Importantly, health-related uncertainty did not exist in isolation but intersected with other domains: it heightened dependence on family caregivers, created additional financial burdens through repeated consultations, and was amplified by social stressors such as hospital-related anxiety and inadequate communication.

### 4.2 Family-related stressors and caregiver support

Family-related stressors emerged as another significant category in this study. The lack of adequate caregiving support from family members, coupled with the emotional strain experienced when caregivers fail to empathize with the patient’s struggles, contributed to additional distress for older women. This finding is consistent with prior research suggesting that the reduced availability of family or friend caregivers for older individuals in the coming decades represents a significant public health concern (35, 36). In such conditions, older patients often feel disappointed by caregiving gaps, particularly when family members are unavailable or unable to provide the emotional support needed.

A novel insight from this study, however, is the prevalence of intergenerational guilt reported by older patients. Many participants expressed guilt for not being able to fulfill traditional family roles, such as assisting with their grandchildren’s education or providing financial support to their children. This phenomenon is particularly pronounced in collectivist societies, such as China, where filial piety is a cultural expectation (37). This finding is particularly significant as it highlights a psychological burden that may not have been fully explored in existing research on chronic illness and family dynamics. While earlier studies have examined family caregiving burdens in chronic illness, this cultural expression of guilt is a context-specific finding and therefore represents an important theoretical extension (38). Previous studies of chronic illness have predominantly examined caregiver burden from younger generations to older parents (39), whereas our findings highlight the reverse dynamic of older women’s guilt toward younger generations, suggesting a culturally specific pattern.

Shortfalls in family caregiving not only aggravated financial stress but also heightened patients’ health-related uncertainty, as limited support forced them to confront illness alone. At the same time, financial insecurity reduced relatives’ capacity to provide care. These overlapping pressures demonstrate how family stressors intersect with health, economic, and social challenges.

### 4.3 Financial stressors and healthcare costs

Financial stressors was also a critical issue for older women with uterine fibroids (40), primarily due to high healthcare costs and limited post-retirement income. Participants without insurance faced considerable difficulties in affording their treatments, often delaying care as a result (41). This financial strain was further compounded by the reliance on fixed incomes after retirement, which made it increasingly difficult to manage rising healthcare expenses.

This finding aligns with existing literature, which indicates that older patients frequently experience financial stress due to high healthcare costs, particularly for those without sufficient insurance coverage (42). The findings suggest that financial challenges for older women with uterine fibroids extend beyond immediate medical expenses, also encompassing long-term financial insecurity. The combination of a reduced fixed income and escalating healthcare costs may restrict access to timely and appropriate treatments, further intensifying chronic stress.

By contrast, studies from high-income countries with universal healthcare systems report that older patients face far fewer barriers to treatment (43), highlighting the structural impact of healthcare financing models on stress experience.

Additionally, another key finding is that financial stress exacerbates both the physical and emotional toll of uterine fibroids. Several participants reported avoiding medical care due to the unaffordable costs, which led to worsened symptoms and a heightened sense of helplessness. This indicates that financial constraints significantly influence the disease trajectory, especially for older adults.

These financial constraints not only restricted medical options, leading to delayed or foregone treatments, but also aggravated family strain as relatives struggled to compensate for limited resources. At the same time, dependence on fragile social support systems left patients vulnerable, underscoring how financial stressors intersect with health, family, and social domains.

### 4.4 Social stressors and support networks

The lack of adequate social support was determined as another significant stressor for older women with uterine fibroids.

While many older women have retired by the time they experience uterine fibroids, a significant number of participants in this study were still employed. For these women, stressors related to job insecurity due to their condition were a major concern. They feared that their symptoms would affect their work performance, potentially leading to job loss. This concern is consistent with existing literature on job insecurity and chronic illnesses (44). However, this study provides a novel insight by highlighting how job insecurity is compounded by the physical limitations of chronic illness. The fear of losing their job due to health problems creates a dual burden for these women, intensifying their stress. It is clear that employers and the broader social system should offer more humanistic care and vocational support to older patients with uterine fibroids, helping to alleviate the chronic pressure faced by this vulnerable group.

The environmental anxiety surrounding hospital visits among older women with uterine fibroids corresponds to the concept of “white coat fear,” as described in previous research (45, 46). Its relevance to older women with fibroids underscores the broader applicability of hospital-induced anxiety across diverse chronic conditions. To address this, hospitals should create more senior-friendly environments by improving accessibility, simplifying administrative processes, and optimizing patient flow.

Existing literature on patient-provider communication emphasizes the critical role of clear, tailored communication in managing chronic conditions (47, 48). This study, however, offers new insight by revealing that uncertainty regarding fibroids persists beyond menopause. Many women continued to experience symptoms without receiving adequate information about their treatment options. This finding underscores the necessity for more individualized care strategies, as the assumption that fibroids naturally shrink post-menopause may not apply to all patients.

Moreover, the results indicate that older patients with uterine fibroids often struggle to understand the medical terminology used by their healthcare providers. While there is an abundance of information available for common chronic diseases like hypertension and diabetes (49), fibroids—despite their high prevalence and substantial disease burden in older women (50)—lack sufficient public awareness. This highlights the need to increase the dissemination of accessible medical information about fibroids to help older patients better understand their condition and its risks.

Many participants felt isolated due to the absence of a supportive social network. Some women reported feeling more at ease when discussing their condition with others who shared similar experiences. Peer support emerged as a vital coping mechanism for many, highlighting the role of informal networks in managing chronic illness. This finding aligns with existing research, which shows that peer support can improve emotional well-being and improve coping strategies among patients with chronic illnesses (51). Additionally, this study introduced the concept of informal education as an effective coping strategy. Participants used online resources, community health lectures, and television programs to educate themselves about their condition, suggesting that informal education could be an important tool for empowering older women with chronic health issues, particularly when formal healthcare support is limited. Unlike in Western contexts where formal support systems are more established (52), our findings indicate that older Chinese women rely heavily on informal peer and online networks, suggesting that cultural and systemic differences shape the forms of social coping available.

Social stressors such as workplace insecurity, hospital anxiety, and information gaps reinforced medical uncertainty, deepened family strain and financial vulnerability, and ultimately amplified psychological distress.

Taken together, the four domains are interrelated: health-related uncertainty increases family strain and financial pressure; family and economic difficulties weaken social support; and social stressors further intensify medical uncertainty and emotional distress. This interplay amplifies the overall stress burden on older women with uterine fibroids.

This study determined four primary dimensions of chronic stressors experienced by older women with uterine fibroids: health-related, family-related, financial, and social stressors. Together, these stressors contribute to significant physical, emotional, and psychological burdens, underscoring the need for a more holistic and patient-centered approach to care. To address these challenges, healthcare providers should offer clearer, tailored information regarding disease progression, treatment options, and symptom management to reduce uncertainty and fears of malignancy. Family-focused strategies are needed to address intergenerational guilt and caregiving gaps, including counseling and family-inclusive care models to alleviate role-related burdens (53). Policymakers must enhance financial assistance programs, particularly for uninsured or low-income older patients (54), to ensure timely access to essential treatment. Social interventions should expand peer support networks, community-based health education programs, and workplace accommodations to reduce job insecurity and strengthen coping capacity. Improving doctor-patient communication and health literacy support also remains a critical component within the social dimension.

By adopting these targeted recommendations, both healthcare providers and society can help ease the chronic stress burden on older women with uterine fibroids, ultimately improving their overall well-being and quality of life.

While this study drew upon Lazarus and Folkman’s stress and coping theory (23) and the biopsychosocial model (24) as guiding frameworks, the findings extend both models in important ways. The stress and coping theory emphasizes individual cognitive appraisal and coping strategies, yet it does not fully capture the influence of culturally embedded stressors, such as intergenerational guilt rooted in filial obligations (55), which were prominent in our participants’ narratives. Similarly, the biopsychosocial model highlights the interplay of biological, psychological, and social dimensions in shaping health outcomes, but it often conceptualizes “social” at a general level, overlooking institutional stressors such as unsupportive hospital environments and gaps in doctor-patient communication (56). By revealing these additional layers of stress, our study suggests that classic models, while valuable, require refinement to account for the sociocultural and institutional contexts that uniquely shape the experiences of older women with uterine fibroids. In doing so, this study contributes to a more comprehensive theoretical understanding of chronic stressors in aging populations, with implications for both cross-cultural research and healthcare system design.

In comparison with other chronic diseases, the stressors observed in this study showed both similarities and differences. Common stressors, such as uncertainty about illness trajectory (57), financial burden (58), and reduced social support (59), are widely reported across chronic illness populations. However, unique patterns emerged in older women with uterine fibroids, including culturally shaped family expectations that foster feelings of guilt (60) and specific challenges linked to unsupportive healthcare settings (61). These findings highlight the need to refine classic stress models by incorporating cultural and systemic dimensions, thereby extending their explanatory power to conditions uniquely shaped by sociocultural contexts.

This study has several limitations that should be considered. First, the participants were recruited from a single tertiary hospital in Shanghai, which may restrict the generalizability of the findings to other regions, healthcare institutions, or community settings. Future research should expand the sample by including multiple sites and more diverse populations. Second, although qualitative content analysis enabled an in-depth understanding of stressors, the findings inevitably reflect subjective interpretations by both researchers and participants. Triangulation with quantitative methods or mixed-methods designs could provide more comprehensive evidence. Third, the study was conducted at one point in time, which does not capture how stressors and coping mechanisms may change as health conditions evolve. Longitudinal studies are needed to explore the dynamic trajectory of stress over time. Finally, some findings, such as intergenerational guilt rooted in filial piety norms, are strongly tied to cultural context. Comparative studies in other cultural environments would help to clarify which stressors are universal and which are specific to particular sociocultural settings.

## 5 Conclusion

This study emphasizes the multifaceted nature of chronic stressors faced by older women with uterine fibroids, revealing significant challenges related to health management, family support, financial assistance, and social support. Health related uncertainty about symptom persistence and fears of malignancy remained major concerns, while family caregiving gaps and intergenerational guilt reflected both practical and cultural burdens. Financial strain, especially for women without adequate insurance or post-retirement income, further limited timely access to care and intensified emotional distress. Social stressors, including workplace insecurity, hospital related anxiety, gaps in doctor–patient communication, and reliance on informal peer networks, compounded these challenges and reinforced feelings of insecurity. Together, these domains formed a mutually reinforcing network that amplified both physical and psychological burdens.

The findings underscore the need for integrated, multidimensional care strategies that combine medical management with psychosocial interventions. Clinicians should provide clearer communication about disease progression and treatment options to reduce uncertainty and fears, while also engaging families in supportive counseling to mitigate caregiving strain and intergenerational guilt. Policymakers should expand financial protection and retirement benefits to reduce economic barriers, and health systems should strengthen community-based peer support and education programs to complement formal care. By addressing these interconnected stressors, the healthcare system can alleviate chronic stress and improve the overall well-being of older women living with uterine fibroids.

Future research should employ longitudinal and mixed methods designs to capture the dynamic trajectory of stress over time and evaluate the effectiveness of targeted interventions. Comparative studies across cultural settings are also warranted to distinguish universal stressors from those shaped by sociocultural and institutional contexts.

## Data Availability

The data used in this study can be obtained by requesting from the corresponding author.
